# Development of a human umbilical cord-derived mesenchymal stromal cell-based advanced therapy medicinal product to treat immune and/or inflammatory diseases

**DOI:** 10.1186/s13287-021-02637-7

**Published:** 2021-11-13

**Authors:** Miryam Mebarki, Nathan Iglicki, Céline Marigny, Camille Abadie, Claire Nicolet, Guillaume Churlaud, Camille Maheux, Hélène Boucher, Antoine Monsel, Philippe Menasché, Jérôme Larghero, Lionel Faivre, Audrey Cras

**Affiliations:** 1 INSERM Centre d’investigation Clinique de Biothérapies CBT501, AP-HP, Hôpital Saint-Louis, Unité de Thérapie Cellulaire, 75010 Paris, France; 2 INSERM U976, Université de Paris, 75010 Paris, France; 3 AP-HP, Hôpital Saint-Louis, Centre MEARY de Thérapie Cellulaire Et Génique, 75010 Paris, France; 4 INSERM UMR1140, Université de Paris, 75006 Paris, France; 5 Faculté de Pharmacie, Université de Paris, 75006 Paris, France; 6 Unité de Soins Intensifs Et Département de Biothérapies, inflammation et immunopathologie, AP-HP, Hôpital La Pitié-Salpêtrière, 75013 Paris, France; 7 INSERM UMR-S 959, Université Sorbonne, 75012 Paris, France; 8 Département de Chirurgie Cardiovasculaire, AP-HP, Hôpital Européen Georges Pompidou, 75015 Paris, France

**Keywords:** Human umbilical cord, Mesenchymal stromal cells, Immunomodulation, Inflammation, Advanced therapy medicinal product, Good manufacturing practice

## Abstract

**Background:**

Umbilical cord-derived mesenchymal stromal cells (UC-MSCs) revealed their key role in immune regulation, offering promising therapeutic perspectives for immune and inflammatory diseases. We aimed to develop a production process of an UC-MSC-based product and then to characterize UC-MSC properties and immunomodulatory activities in vitro, related to their clinical use and finally, to transfer this technology to a good manufacturing practice (GMP) compliant facility, to manufacture an advanced therapy medicinal product (ATMP).

**Methods:**

Fifteen human umbilical cords (UCs) were collected to develop the production process. Three batches of UC-MSCs from a single donor were characterized at basal state and after in vitro pro-inflammatory stimulation by interferon-γ (IFNγ) and tumor necrosis factor-α (TNFα). Proliferation, immunophenotype, activation markers’ expression and the inhibition of T cell proliferation were assessed. Finally, this technology was transferred to a GMP-compliant facility to manufacture an UC-MSC-based ATMP, from a single donor, using the explant method followed by the establishment of master and work cell stocks.

**Results:**

Twelve UCs were processed successfully allowing to isolate UC-MSCs with doubling time and population doubling remaining stable until passage 4. CD90, CD105, CD73, CD44, CD29, CD166 expression was positive; CD14, CD45, CD31, HLA-DR, CD40, CD80 and CD86 expression was negative, while CD146 and HLA-ABC expression was heterogeneous. Cell morphology, proliferation and immunophenotype were not modified by inflammatory treatment. Indoleamine 2,3-dioxygenase (IDO) expression was significantly induced by IFNγ and IFNγ + TNFα *versus* non-treated cells. Intercellular adhesion molecule-1 (ICAM-1) and vascular cell adhesion molecule 1 (VCAM-1) expression was induced significantly after priming. T cell proliferation was significantly decreased in the presence of UC-MSCs in a dose-dependent manner. This inhibitory effect was improved by IFNγ or IFNγ + TNFα, at UC-MSCs:PBMC ratio 1:10 and 1:30, whereas only IFNγ allowed to decrease significantly T cell proliferation at ratio 1:100. The manufacturing process of the UC-MSC-based ATMP was qualified and authorized by the French regulatory agency for clinical use (NCT04333368).

**Conclusion:**

This work allowed to develop an investigational UC-MSC-based ATMP authorized for clinical use. Our results showed that an inflammatory environment preserves the biological properties of UC-MSCs with an improvement of their immunomodulatory functions.

**Supplementary Information:**

The online version contains supplementary material available at 10.1186/s13287-021-02637-7.

## Background

Mesenchymal stromal cells (MSCs) emerge as a perspective for the development of advanced therapy medicinal products (ATMPs). Bone marrow (BM) was proposed as the first source, autologous or allogeneic, to obtain MSCs [1]. Bone marrow-derived MSCs (BM-MSCs) have been described to participate in homeostasis and tissue repair and are thus investigated as tissue-engineered products in the field of regenerative medicine [2]. Later, umbilical cord-derived MSCs (UC-MSCs) have been described to display high immunomodulatory and anti-inflammatory properties [3], which has attracted attention for their use in the treatment of immune and/or inflammatory disorders. Indeed, they have been reported to modulate the immune system in multiple diseases such as graft *versus* host disease (GvHD) [4] and systemic lupus erythematous [5]. Moreover, UC-MSCs are weakly immunogenic, thus completing the MSCs safety profile that was demonstrated in several clinical trials [6]. Finally, sourcing MSCs from umbilical cord (UC) raises less technical and ethical issues compared to BM. Thus, UC-MSCs are considered promising candidates to develop MSC-derived ATMPs [7].

Since the publication of the European Directive 2003/63/EC and the European Regulation 1394/2007/EC, MSC-based products are classified as ATMPs if MSCs have been subjected to substantial manipulation so that their biological characteristics, physiological functions or structural properties, relevant for the intended clinical use, have been modified or if they are not intended to be used for the same essential function(s) in the recipient and the donor. To date, most of the MSC-based ATMPs are composed of BM-MSCs or adipose tissue-derived MSCs (AT-MSCs). As an example, Prochymal® and Temcell® are cell therapy medicinal products composed of BM-MSCs. Alofisel®, the only MSC-derived ATMP authorized in Europe is composed of AT-MSCs. The European Medicines Agency (EMA) has delivered recommendations to classify more than sixty UC-MSC-based products as ATMPs [8]. However, no UC-MSCs candidate product is currently under evaluation for marketing authorization (MA).

The aims of our project were first to develop and validate a process of MSCs isolation from human UCs and their expansion in vitro and second to investigate the biological characteristics of the obtained UC-MSCs at their basal state and after inflammatory challenge to provide the rationale of using UC-MSCs for their immunomodulatory functions, in immune and/or inflammatory diseases. Indeed, several studies have shown that the immunomodulatory capacities of MSCs are not constitutive, but rather driven by the pro-inflammatory cytokines secreted by antigen-presenting cells and T cells, including interferon-γ (IFNγ), tumor necrosis factor-α (TNFα) and interleukin (IL) 1β [9, 10]. The inflammatory status of patients was simulated by an in vitro treatment with these cytokines. In addition, we evaluated the impact of two supplementary cytokines, IL6 largely described as a mediator of inflammation and autoimmunity, and granulocyte macrophage colony-stimulating factor (GM-CSF) involved in chronic inflammation [11, 12]. Finally, once the UC-MSC properties have been confirmed, this technology was transferred to a good manufacturing practice (GMP)-compliant facility to manufacture an investigational UC-MSC-based ATMP for clinical use in the treatment of severe acute respiratory syndrome coronavirus-2 (SARS–CoV-2)-induced acute respiratory distress syndrome (ARDS) [13]. This process has been authorized by the French regulatory agency.

## Material and methods

### UC-MSCs’ isolation and expansion process

#### UC collection

UCs were collected by the Cell Therapy Unit, Saint Louis Hospital (AP-HP, Paris, France) from maternities affiliated to the Allogeneic Cord Blood Bank (CBB), coordinated by the French Placental Blood Network of French Biomedicine Agency (ABM). The UC collection process was approved by the French regional health agency (ARS, Ile de France). Donation, procurement, testing, processing and storage were performed in accordance with the European Directive 2004/23/EC. UCs were collected from 15 healthy donors, who signed up a fully informed donor consent. Serological tests were performed according to the European directive on tissue procurement (Additional file 1).

#### UC-MSC isolation and expansion

UC-MSCs were isolated from UC according to the explant method [14]. Using a sterile surgical scalpel, the UC was dissected longitudinally, blood vessels were removed and the Wharton’s jelly was scratched. The UC was cut into fragments of few centimeters named explants and then seeded in one or several 6 well plates, in a qualified complete culture medium composed of NutriStem® MSC XF Basal Medium (Biological Industries, Ref 05-200-1A) + Nutristem® MSC XF Supplement Mix (Biological Industries, Ref 05-200-1U) + 5% irradiated platelet lysate (PL) MultiPL100’i (Macopharma, Ref BC0190032) + sodium Heparin 2 IU/mL (Panpharma, Ref 5520508). The UC was maintained at 37 °C, in a humidified atmosphere with 5% CO_2_, and culture medium was changed twice a week. During the whole process, cell confluence and morphology were assessed in situ, using a phase-contrast microscope. At day 7, the UC was removed and UC-MSCs were cultured until reaching colonies with 80% confluence (passage 0). UC-MSCs were harvested using a recombinant trypsin EDTA solution (Biological Industries, Ref 03-079-1B) and then seeded for further expansions in a higher surface culture, for one or several passages, until a suitable cell quantity was reached.

### Characterization of UC-MSCs’ biological properties and functions

#### Cell proliferation assessment

UC-MSCs were enumerated after each passage using a manual Malassez counting Chamber. Doubling time (DT) and population doubling (PD) were determined after each passage according to the formulas (T × log(2))/(log Y − log X) and (Y/X)/log(2), respectively (X: number of cells originally seeded, Y: number of cells harvested at passage and T: time of culture in hours).

#### Cell size measurement

UC-MSCs’ size was measured using the Incucyte® S3 and analyzed on the Incucyte® S3 Live—Cell Analysis system (Sartorius). The measures were performed on 3 batches and 4 microscopic fields per batch.

#### Priming with pro-inflammatory cytokines

In order to assess the immunomodulatory properties of UC-MSCs, we created in vitro an inflammatory environment mimicking that seen in patients. UC-MSCs were treated with the pro-inflammatory cytokines IFNγ, TNFα, IFNγ + TNFα, IL1β, IL6, GM-CSF and a mix of all cytokines (Additional file 2, at the concentration of 10 ng/mL each, during 48 h). A not-treated (NT) condition was used for basal state. After priming, cells were harvested using a recombinant trypsin EDTA solution (Biological Industries, Ref 03-079-1B), washed and suspended in the adequate medium depending on the analysis.

#### UC-MSCs’ phenotype

UC-MSCs phenotype was assessed at basal state and under pro-inflammatory challenge conditions. Cells were suspended in 100 µL PBS/albumin 1% and stained with a panel of antibodies (Additional file 3), for 15 min at 4 °C, protected from light. A titration was performed to determine the optimal concentration of each antibody. The following dilutions were tested: 1/50 (recommended by the supplier), 1/100, 1/200, 1/400, 1/800 and 1/1600 for anti-HLA-ABC, anti-CD86, anti-CD31 and 1/20, 1/40, 1/80, 1/160, 1/320 and 1/640 for others. Cells were washed in 1 ml PBS/Albumin 1% and centrifuged at 1500 RPM for 5 min. After the removal of supernatant, cells were suspended in 300 µL PBS/albumin 1%. Negative controls were non-stained cells or Fluorescence Minus One for CD31, CD14 and CD45 antibodies. The acquisitions were performed on an Attune NxT™ ThermoFisher® Flow Cytometer, and analyses were performed using Attune NxT software.

#### Potency assays

To assess the immunomodulatory properties of UC-MSCs, we performed, as potency assays, the following two assays according to the International Society for Cell & Gene Therapy (ISCT®) recommendations [15].

##### Activation markers’ expression

Activation markers’ expression was evaluated on non-treated UC-MSCs (NT) and after priming by pro-inflammatory cytokines for 48 h. For Indoleamine 2,3-dioxygenase (IDO) staining, UC-MSCs were suspended in 100 µL PBS/albumin 0.1% and stained with 2 µL of human anti-CD90 FITC antibody for 15 min, at room temperature. After washing in PBS/Albumin 0.1%, cells were fixed with an intracellular fixation buffer for 60 min, at room temperature and then permeabilized twice with permeabilization buffer (eBioscience, Ref 88,882,400). Cells were suspended in 100 µL of permeabilization buffer and labeled with 5 µL of human anti-IDO e-Fluor-660 antibody (eBioscience, Ref 50947742) for 20 min at room temperature and washed in the permeabilization buffer and then in PBS/albumin 0.1%.

The intercellular adhesion molecule-1 (ICAM-1/CD54), programmed death ligand 1 (PD-L1/CD274), vascular cell adhesion molecule 1 (VCAM-1/CD106), CD200, INFγ-Receptor (INFγ-R/CD119) and TNFα-Receptor II (TNF-RII/CD120b) were assessed at basal state (NT) and after pro-inflammatory treatment according to the protocol described above (Additional file 3). Acquisitions were performed with an Attune NxT™ ThermoFisher® Flow Cytometer and analyses using Attune NxT™ software.

##### Mixed lymphocyte reaction (MLR)

MLR potency assay was performed according to Nicotra et al. [16]. MLR assay was performed on UC-MSCs both in a resting state (NT) and after priming for 48 h with IFNγ, TNFα and IFNγ + TNFα. Briefly, peripheral blood mononuclear cells (PBMCs) pooled from 10 healthy donors and labeled with the CellTrace^TM^ Violet (CTV) Cell Proliferation Kit (Invitrogen, Ref C34557) were co-cultured with UC-MSCs at 0:1 (control), 1:10, 1:30, 1:100, 1:300 and 1:1000 UC-MSCs:PBMC ratio with a constant amount of PBMC (3 × 10^5^) and a decreasing amount of UC-MSCs from 3 × 10^4^ down to 3 × 10^2^.

Cells were co-cultured in a culture medium composed of Roswell Park Memorial Institute (RPMI) Medium 1640, GlutaMAX^TM^ Supplement, HEPES (Gibco, Ref 72400-013), 10% human A/B serum (Eurobio, Ref CAEHUM010U), 1% Amphotericin B/Penicillin/Streptomycin (Gibco, Ref 15240062), and 10 UI/mL Heparin (PanPharma, Ref 5520508) at 37 °C, 5% CO_2_ for 7 days. At day 4 ± 1, 50 µL of culture medium was added. At day 7, cells were labeled with 5 µL of human antibodies anti-CD3 PE (BD Biosciences, Ref 345765), anti-CD45 FITC (BD Biosciences, Ref 345808) and 7-AAD viability dye (Beckman Coulter, Ref A07704) for 15 min at 4 °C, protected from light. Cells were washed in PBS 1X before acquisition on the Attune NxT™ Thermofisher® Flow Cytometer and analyzed using Attune NxT™ software.

### Manufacturing process of UC-MSC-based investigational ATMP batches

The manufacturing of each UC-MSCs batch was performed from the UC of a single donor, under GMP conditions by the MEARY Cell and Gene Therapy Center at Saint-Louis Hospital (Paris, France) and according to the process developed and validated by the Cell Therapy Unit (CTU) of Saint-Louis Hospital. From the collection of the UC up to the final batch of UC-MSCs for clinical use, the process was divided into two steps. First, according to the European Directive 2004/23/EC, UC receipt and qualification followed by UC-MSCs isolation and derivation of the Master Cell Stock (MCS) were performed at the CTU. The MCS was then transferred to the MEARY center for GMP production of the Work Cell Stock (WCS) and the clinical trial batches, using qualified and certified raw materials and equipment according to the GMP specific to ATMPs [17]. The whole manufacturing process is described in Additional file 4.

#### Master cell stock (MCS)

Upon receipt at the CTU, the collected UC was washed in Gentamicin 0.2 mg/mL (Panpharma, Ref 3512031) before further processing using the explant method as described above. The full UC was seeded in a 150-cm^2^ culture flask (Corning, Ref 90552) in complete culture medium, until passage 0. At day 11, UC-MSCs were harvested using a recombinant trypsin EDTA solution (Biological Industries, Ref 03-079-1B) and then seeded in a 175-cm^2^ culture flask (Corning, Ref 353112) at a density of 500 cells/cm^2^ and expanded until passage 1 (P1). At day 17, UC-MSCs were harvested and seeded in 40 × 672 cm^2^ Cellstack® (Corning, Ref CE0459) at a density of 2000 ± 1000 cells/cm^2^ and expanded until P2, under the same conditions as for P1. At day 21, cells were harvested, formulated for freezing in 50% Dulbecco’s phosphate-buffered saline (DPBS) (Macopharma, Ref BC0120030) + 40% albumin 50 g/L (Octapharma, Ref 575080-0) + 10% Dimethylsulfoxide (DMSO) (Wak Chemie, Ref USP9A1S) and filled into four cryobags (CryoMacs, ref 200-074-401, Miltenyi Biotec) containing 60 × 10^6^ UC-MSCs/bag and then cryopreserved in a vapor nitrogen tanker as a MCS.

#### Work cell stock (WCS)

Upon transfer to the MEARY center, each MCS cryobag was thawed and UC-MSCs were expanded in the complete culture medium in 25 × 672 cm^2^ Cellstack® at a density of 4000 ± 1000 cells/cm^2^ until P3, under the same culture conditions as for P1 and P2 described above. During the whole process, cell confluence and morphology were assessed using an in situ phase-contrast microscope. At day 6 ± 1, cells were harvested, filled into cryobags at the concentration of 100 × 10^6^ UC-MSCs/bag, frozen and cryopreserved as a WCS until need for clinical use to treat immune and/or inflammatory diseases.

#### Manufacture and formulation of the final UC-MSC-based ATMP

For a clinical use, we validated the following protocol to manufacture and formulate the final investigational ATMP. The day of patient injection, a WCS bag will be thawed in a dry bath at 37 °C for 2–3 min, and then, cells will be washed in 0.9% NaCl (Fresenius, 367512-9) + 0.5% albumin (Octapharma, Ref 575080-0). The final medicinal product will consist of a cell suspension in a final volume of 150 mL 0.9% NaCl + 0.5% albumin and will contain 100 × 10^6^ UC-MSCs allowing each bag to deliver a dose of 1 × 10^6^ cells/kg. The ATMP will be transferred to the hospital pharmacy unit at 22 ± 2 °C, before distribution to the clinical department within 4 h.

#### Quality controls (QCs)

In order to qualify and validate the manufacturing process and owing to the limited shelf-life of the final reconstituted ATMP, QCs were performed at the MCS and WCS steps to allow releasing the final investigational medicinal product without any delay.

##### Cell counting and viability

UC-MSCs were enumerated after each passage using two methods: a manual Malassez counting chamber and the automated NucleoCounter NC-200™ (ChemoMetec). Viability was assessed using the automated NucleoCounter NC-200™. In addition, cells’ clonogenicity was assessed using the colony-forming unit-fibroblastic (CFU-F) assay.

##### Immunophenotype

UC-MSCs’ phenotype was evaluated by flow cytometry (MacsQuant10, Miltenyi). The surface antigens CD105, CD73, CD90, CD45, CD34, CD11b, CD19 and HLA-DR were used (hMSC analysis kit, BD biosciences, ref 562245). Viability was assessed using eBioscience Fixable Viability Dye eFluor 780 (Invitrogen, ref 65-0865).

##### Sterility assays

All sterility assays were performed according to the European Pharmacopeia 10th edition. Aerobic and anaerobic BacT/ALERT® tests (Biomérieux) were used and analyzed on the BacT/ALERT® lecturer. Mycoplasmas were quantified using the Venor® GeM qEP test (Minerva Biolabs) on the QuantStudio5 Real-Time PCR System (ThermoFisher Scientific) and endotoxins using the Chromo-LAL test (Associates of Cape Cod) on the Multiskan Sky Spectrophotometer (ThermoFisher Scientific).

##### Karyotype

Karyotype was performed according to the International Organization for Standardization ISO 15189, on a minimum of 20 metaphases. Briefly, cells were blocked in metaphase by colchicine; chromosomes were dispersed by hypotonic shock and fixed by alcohol and acetic acid. Different banding techniques were used to obtain a specific staining of each chromosome. Mitoses were captured on a software and chromosomes classified by pair.

##### Potency assay

MLR potency assay was performed as described above. T cell proliferation was calculated using the area under the curve (AUC) as previously described [16].

### Statistical analysis

Statistical analyses were performed using GraphPad PRISM® 8.4.0 software with appropriate tests as specified in the respective results section below. Descriptive data are expressed as mean [min–max], and all other values are expressed as mean ± standard deviation. A minimum of 95% confidence interval was established for significance. A *p* value < 0.05 was considered statistically significant. Kruskal–Wallis, Bonferroni’s and Dunnett's tests were used, as appropriate.

## Results

Before considering the use of UC-MSCs for clinical indications, it was necessary to design a process allowing to isolate and expand the MSCs collected from human UC units as an infinite and easily accessible source of the starting material.

### Development of the production process

**Fig. 1:**
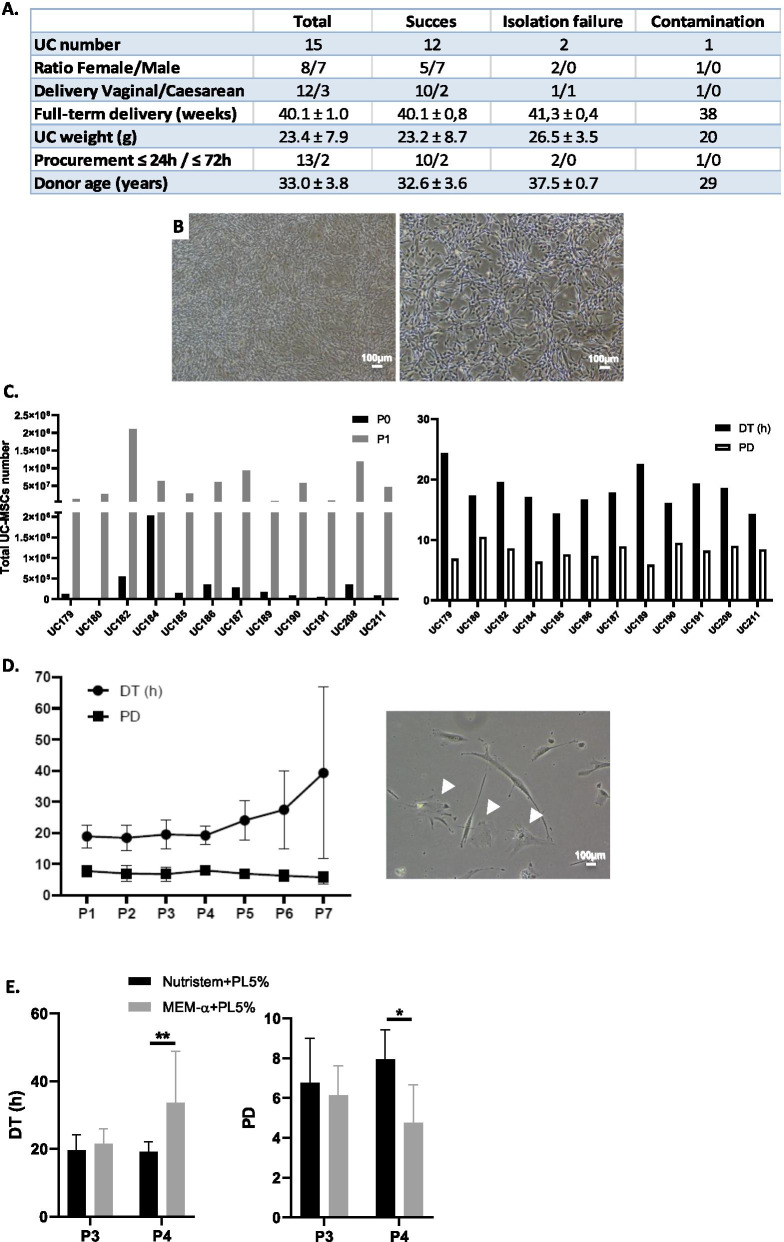
Development of UC-MSCs’ production process. **A** Characteristics of collected and processed UCs (*n* = 15 UCs). **B** UC-MSCs morphology at passage 1 (P1) at 4X (left) and 10X (right) magnifications. **C** UC-MSCs quantity at P0 and P1 (left) and DT (h) and PD calculated from P0 to P1 (right) for each UC (*n* = 12 UCs). **D** DT (h) and PD from P1 to P7 (*n* = 4 UCs) (left) and UC-MSCs morphology at P7 (right) representing senescent morphology (head arrows). **E** Comparison of DT (h) and PD between culture media composed of Nutristem® + PL5% and MEM-α + PL 5% at P3 and P4 (*n* = 4 UCs)

Among fifteen collected UCs, twelve (80.0%) were processed successfully, allowing to isolate UC-MSCs. There were three failures to process UC-MSCs, of which two (13.3%) were due to cell isolation failure and one (6.7%) to bacterial contamination during the culture (Fig. 1A). Isolated cells were elongated and thin, exhibited a fibroblast-like morphology and presented a size of 201.4 ± 57.0 µm (Fig. 1B).

The mean total UC-MSCs quantity isolated per UC was 3.6 × 10^5^ [0.2 × 10^5 ^− 20.3 × 10^5^] corresponding to a yield of 15.1 × 10^3^ [1.3 × 10^3 ^− 81.3 × 10^3^] UC-MSCs per gram of UC (*n* = 12), which was not correlated to the age of donors (*R* = 0.56, *p* > 0.05, Spearman test, data not shown). We defined this step as passage 0 (P0). After expansion at P1, total cell quantity was 6.6 × 10^7^ [0.7 × 10^7 ^− 21.0 × 10^7^]; DT (h) and PD between P0 and P1 were 18.2 [14.3 − 24.4] and 8.1 [5.9 − 10.5], respectively. Cell quantity, DT and PD are described for each UC in Fig. 1C.

Then, we assessed the long-term proliferative capacities of UC-MSCs. DT and PD were evaluated until P7 (*n* = 4). DT and PD were stable until P4 and were, respectively, 18.4 ± 4.2 h and 7.0 ± 2.5 at P2, 19.5 ± 4.6 h and 6.8 ± 2.2 at P3, 19.2 ± 3.0 h and 7.9 ± 1.5 at P4. From P5, DT increased and were more variable, whereas PD decreased yielding values of 24.1 ± 6.3 h and 6.9 ± 1.3 at P5, 27.5 ± 12.5 h and 6.3 ± 1.8 at P6, 39.2 ± 27.6 h and 5.8 ± 2.0 at P7, respectively (*p* > 0.05, Kruskal–Wallis test; Fig. 1D). At later passages, enlarged and flattened cells corresponding to a senescent morphology have been observed (Fig. 1D).

In order to validate the GMP-enriched culture medium Nutristem® used in association with 5% PL (Nutristem® + 5% PL), a comparison with the standard basal medium minimum essential medium alpha (MEM-α) with 5% PL (MEM-α + 5% PL) was performed. Considering the data above, this analysis was focused on P3 and P4 (*n* = 4). DT (h) and PD were similar at P3 (Fig. 1E). At P4, DT was significantly shorter and less heterogeneous with Nutristem® + 5% PL medium compared to MEM-α + 5% PL (19.2 ± 4.6 h *vs* 33.7 ± 15.1 h; *p* < 0.01), whereas PD was significantly higher (7.9 ± 1.5 versus 4.8 ± 1.9; *p* < 0.05) (Bonferroni’s test, Fig. 1E).

Based on these results, P3 was determined as the highest passage number acceptable for clinical use. To confirm the cell safety at this passage level, we performed a karyotype analysis at P3. No chromosomal abnormalities were detected with 46 XY karyotypes for male UCs and 46 XX for female.

### Biological properties and immunomodulatory functions of UC-MSCs

To characterize UC-MSCs, we evaluated their biological properties on three batches of an UC collected from a single donor, which was used to transfer the manufacturing process to a GMP-compliant facility, for clinical use. Analyses were performed after cell thawing at P3.

#### UC-MSCs immunophenotype at basal state

**Fig. 2:**
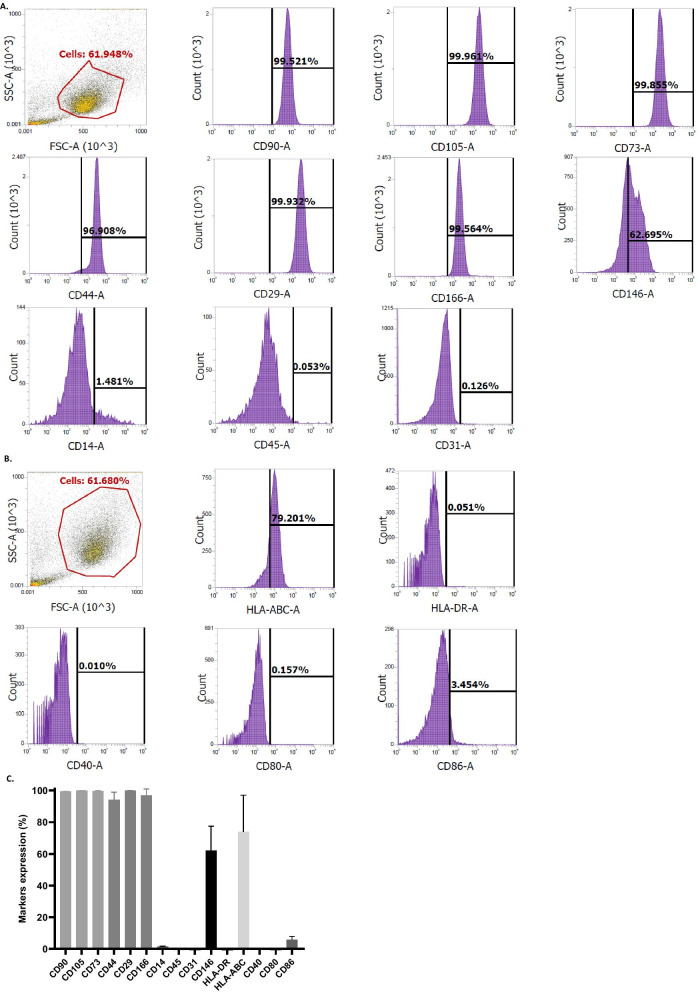
UC-MSCs immunophenotype at basal state. **A** Expression of mesenchymal markers (CD90, CD105, CD73), adhesion molecules (CD44, CD29, CD166, CD146), hematopoietic (CD14, CD45) and endothelial (CD31) markers. **B** Expression of immunogenic (HLA-ABC, HLA-DR) and co-stimulatory (CD40, CD80, CD86) markers. **C** Summary of markers’ expression represented as mean ± standard deviation (%) (*n *= 3 batches from 1 UC)

UC-MSCs expressed positively the mesenchymal markers described by the ISCT® CD90, CD105 and CD73 (99.5 ± 0.1%; 99.9 ± 0.1%; 99.7 ± 0.3%), and adhesion molecules CD44, CD29 and CD166 (94.2 ± 4.7%; 99.9 ± 0.0%; 96.8 ± 4.1%) (Fig. 2A, C). Interestingly, the expression of the adhesion molecule CD146 was moderately positive (62.0 ± 15.5%) showing a heterogeneity between cell subsets (Fig. 2A, C). The expression of hematopoietic markers CD14 and CD45 as well as of the endothelial marker CD31 was negative (1.5 ± 0.2%; 0.1 ± 0.1% and 0.2 ± 0.1%, respectively).

Immunogenic human leukocyte antigen HLA-ABC class I was positively expressed (73.9 ± 23.3%) by UC-MSCs; conversely, the cells did not express HLA-DR class II (0.1 ± 0.1%) or co-stimulatory molecules CD40, CD80 and CD86 (0.0 ± 0.0%; 0.2 ± 0.1% and 5.9 ± 2.0%, respectively) (Fig. 2B, C).

Negative controls are presented in Additional file 5.

#### UC-MSCs’ characteristics after pro-inflammatory priming

To assess whether the pro-inflammatory environment encountered in immune and inflammatory diseases could impact the biological properties of UC-MSCs after their administration, we treated cells for 48 h with several pro-inflammatory cytokines in vitro*.*

**Fig. 3:**
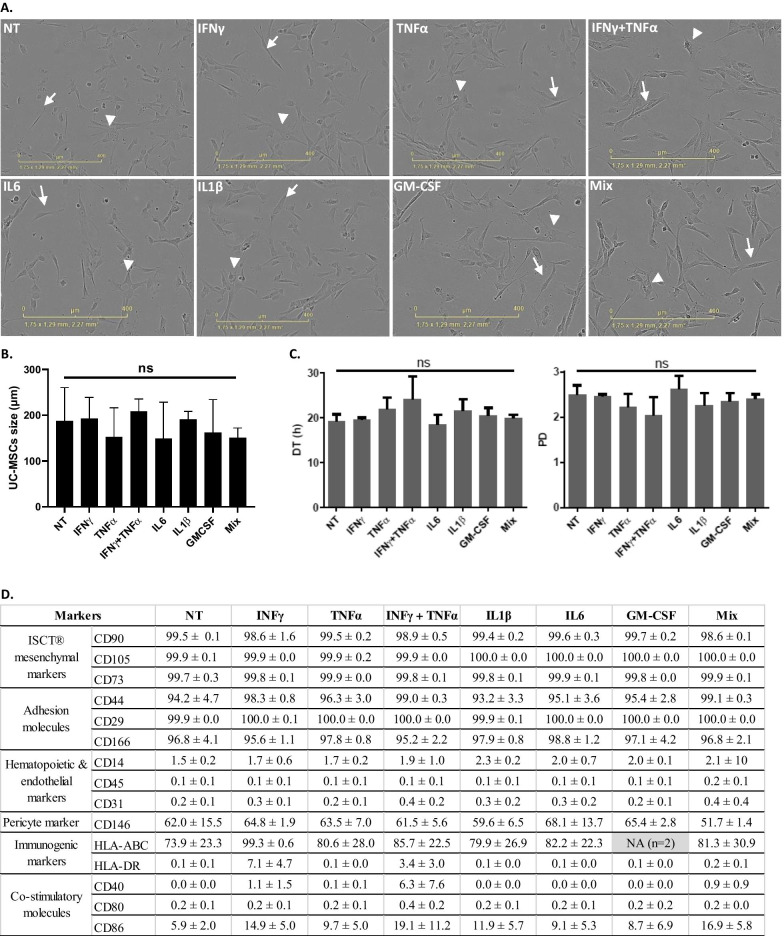
UC-MSCs’ characteristics after pro-inflammatory priming. **A** UC-MSCs’ morphology; **B** UC-MSCs’ size (µm); **C** DT (h) (left) and PD (right); and **D** UC-MSCs’ immunophenotype at basal state (NT) and after pro-inflammatory treatment by IFNγ, TNFα, IFNγ + TNFα, IL6, IL1β, GM-CSF and Mix. NA: Not applicable. *n* = 3 batches from 1 UC

After inflammatory stimulation, cell morphology described above was not modified with the presence of a majority of long and thin cells (Fig. 3A, white arrows), which corresponds to viable cells, and rare enlarged and flattened cells (Fig. 3A, head arrows). Cell size was not significantly influenced either, with an average size of 205.0 ± 64.2 µm for IFNγ; 170.7 ± 43.6 µm for TNFα; 215.3 ± 50.2 µm for IFNγ + TNFα; 168.7 ± 60.7 µm for IL6; 180.2 ± 36.6 µm for IL1β; 178.5 ± 48.7 µm for GM-CSF and 148.5 ± 34.8 µm for mix comparing to 201.4 ± 57.0 µm for NT cells (*p* > 0.05, Kruskal–Wallis test, Fig. 3B).

DT and PD were, respectively, 19.5 ± 0.5 h and 2.5 ± 0.05 for IFNγ; 21.9 ± 2.6 h and 2.2 ± 0.3 for TNFα; 24.1 ± 5.0 and 2.0 h ± 0.4 for IFNγ + TNFα; 18.5 ± 2.1 h and 2.6 ± 0.3 for IL6; 21.5 ± 2.6 h and 2.3 ± 0.3 for IL1β; 20.5 ± 1.7 h and 2.3 ± 0.2 for GM-CSF; 19.9 ± 0.7 h and 2.4 ± 0.1 for Mix *versus* 19.3 ± 1.5 h and 2.5 ± 0.2 for NT cells (Fig. 3C). Thus, the pro-inflammatory treatment did not influence the proliferative capacities of cells regardless of the used cytokine (*p* > 0.05; Kruskal–Wallis test). In addition, no significant difference was observed in the tested cell markers (*p* > 0.05; Kruskal–Wallis test), thereby showing that a simulated inflammatory environment does not affect the UC-MSCs’ immunophenotype (Fig. 3D).

#### UC-MSCs’ biological activity in vitro after pro-inflammatory priming

##### Activation markers expression

**Fig. 4:**
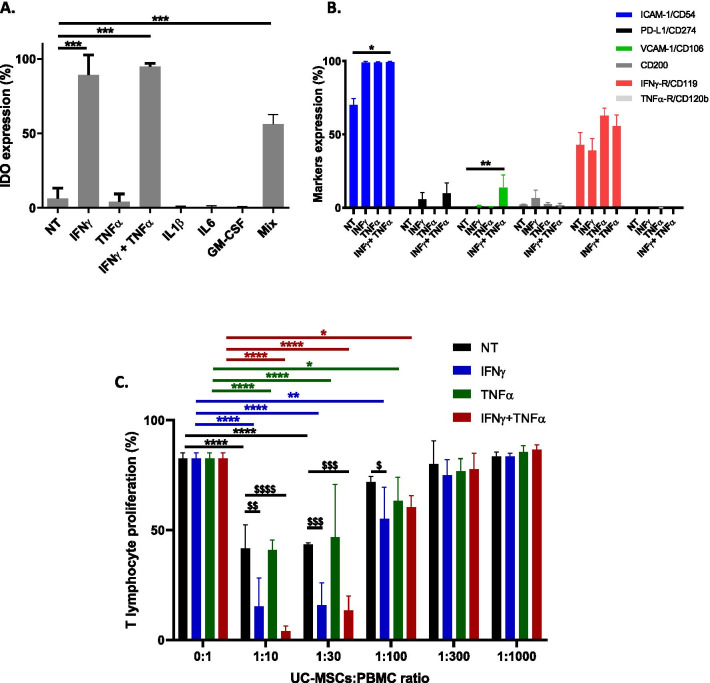
UC-MSCs’ biological activity in vitro after pro-inflammatory priming. **A** IDO expression (%) at basal state (NT) and after pro-inflammatory priming by IFNγ, TNFα, IFNγ + TNFα, IL1β, IL6, GM-CSF and Mix. **B** ICAM-1/CD54, PD-L1/CD274, VCAM-1/CD106, CD200, IFNγ-R/CD119, TNFα-RII/CD120b expression (%) at basal state (NT) and after pro-inflammatory priming by IFNγ, TNFα and IFNγ + TNFα. **C** T-lymphocyte proliferation (%) at ratio UC-MSCs:PBMC 0:1, 1:10, 1:30, 1:100, 1:300 and 1:1000 at basal state (NT) and after pro-inflammatory priming by IFNγ, TNFα and IFNγ + TNFα. */^$^*p* < 0.05, **/^$$^*p* < 0.01, ***/^$$$^*p* < 0.001, ****/^$$$$^*p* < 0.0001. *n* = 3 batches from 1 UC

To evaluate UC-MSCs’ immunomodulatory properties, we first assessed the IDO expression, a key molecule involved in the inhibition of T cell proliferation [18]. IDO expression was significantly induced by IFNγ (85.0 ± 10.8%) and IFNγ + TNFα (90.1 ± 7.7%) comparing to the NT group (6.2 ± 7.0%) (*p* < 0.001, Dunnett’s test, Fig. 4A). However, pre-treatment with TNFα, IL1β, IL6 or GM-CSF did not influence IDO expression. When cells were primed with all pro-inflammatory cytokines (Mix), we observed an increase of IDO expression (56.2 ± 6.5%, *p* < 0.001) underlying the key role of IFNγ to induce IDO expression.

As MSCs’ immunomodulatory functions involve cell–cell interactions, we assessed the expression of cell surface markers such as immune and adhesion molecules as well as cytokine receptors including ICAM-1/CD54, PD-L1/CD274, VCAM-1/CD106, CD200, IFNγ-R/CD119 and TNFα-RII/CD120b. Our results showed that UC-MSCs expressed moderately ICAM-1/CD54 (70.1 ± 7.6%) and IFNγ-R/CD119 (42.7 ± 15.1%) at basal state (Fig. 4B). After pro-inflammatory treatment, ICAM-1 expression increased to 99.2 ± 1.0% with IFNγ, 99.0 ± 1.1% with TNFα and 99.3 ± 1.0 with IFNγ + TNFα (p < 0.05, Kruskal–Wallis test). Interestingly, the expression of VCAM-1/CD106 was negative at basal state, but increased significantly after priming with IFNγ + TNFα to 13.5 ± 15.3% (*p* < 0.05, Kruskal–Wallis test, Fig. 4B). Other molecules were not expressed either at basal state or under pro-inflammatory conditions.

##### Inhibition of T-lymphocyte proliferation

To confirm the immunomodulatory properties of UC-MSCs, we performed a potency assay using the MLR assay according to ISCT recommendations [15], at basal state and after treatment with IFNγ, TNFα and IFNγ + TNFα. To assess whether the inhibitory effect of UC-MSCs on T-lymphocyte (T-Ly) proliferation is dose dependent, we tested several ratios of UC-MSCs:PBMCs co-culture. At basal state, T-Ly growth was inversely proportional to UC-MSCs quantity (Fig. 4C). The inhibition of T-Ly proliferation was significant for ratio 1:10 and 1:30 with proliferation rates of 41.7% and 43.4%, respectively, compared to 82.6% in the absence of UC-MSCs (p < 0.0001, Dunnett's test, Fig. 4C). Pro-inflammatory treatment with IFNγ, TNFα and IFNγ + TNFα inhibited T-Ly proliferation up to the 1:100 ratio, demonstrating the enhancement of UC-MSCs activity even at very low cell concentrations. Thus, T-Ly proliferation rates at 1:10, 1:30 and 1:100 ratios were decreased as follows: for IFNγ: 15.3% (*p* < 0.0001), 15.7% (*p* < 0.0001) and 55.1% (*p* < 0.01), respectively; for TNFα: 40.8% (*p* < 0.0001), 46.7% (*p* < 0.0001) and 63.3% (*p* < 0.05), respectively; and for IFNγ + TNFα: 3.9% (*p* < 0.0001), 13.5% (*p* < 0.0001) and 60.3% (*p* < 0.05), respectively.

Interestingly, T-Ly proliferation was significantly decreased after priming by IFNγ or IFNγ + TNFα compared to basal state, at 1:10 (*p* < 0.01 and *p* < 0.0001) and 1:30 ratios (*p* < 0.001 for both), whereas only IFNγ allowed to decrease significantly T-Ly proliferation at the 1:100 ratio (*p* < 0.05) (Dunnett's test, Fig. 4C). However, there was no significant difference in the decrease of T-Ly proliferation between IFNγ or IFNγ + TNFα (*p* > 0.05) regardless of the UC-MSCs:PBMCs ratio.

### Transfer of the manufacturing process to a GMP-compliant facility

Characterization of the biological properties and immunomodulatory functions of UC-MSCs derived from the production process developed by the CTU confirmed that the process was fully operational. It was thus transferred to the MEARY Cell and Gene Therapy Center, GMP-compliant site for routine production of an investigational UC-MSC-based ATMP.

**Fig. 5:**
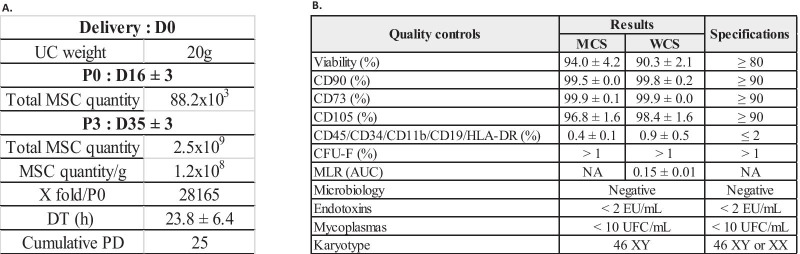
Qualification of the UC-MSC-based ATMP manufacturing process. **A** Quantities and proliferation data of UC-MSCs from P0 to P3. **B** Quality controls performed during MCS (*n* = 2 batches) and WCS steps (*n* = 3 batches)

An UC weighing 20 g was collected from a single healthy donor. At P0, 88.2 × 10^3^ cells were isolated using the explant method. After expansion, 4.6 × 10^7^ UC-MSCs were obtained at P1 and 2.2 × 10^8^ at P2, that were cryopreserved in four bags constituting the MCS. Two bags were thawed, and cells were amplified until P3, allowing to obtain 2.5 × 10^9^ UC-MSCs, which were cryopreserved in 26 bags corresponding to the WCS. At this step, 1.2 × 10^8^ UC-MSCs were obtained per gram of UC after three passages, with a 28 165-fold expansion from P0 to P3. The mean DT (h) was 23.8 ± 6.4 and the cumulative PD was 25 (Fig. 5A). To validate the manufacturing process, QCs were performed including cell viability, immunophenotype and CFU-F on cells from the MCS (*n* = 2 batches) and WCS (*n* = 3 batches) and a MLR assay for those of the WCS (*n* = 3 batches). Viability was 94.0 ± 4.2% for MCS and 91.0 ± 1.6% for WCS and was compliant for all batches (≥ 80%). CD90, CD73 and CD105 markers’ expression was ≥ 90%, whereas CD45, CD34, CD11b, CD19 and HLA-DR was ≤ 2%, according to the defined specifications (Fig. 5B). The CFU-F assay showed UC-MSCs clonogenicity (> 1%). Finally, MLR assay showed an AUC of 0.15 ± 0.01 for the WCS, confirming UC-MSCs immunomodulatory properties. Safety assessment showed normal karyotype (46 XY), negative microbiology, mycoplasmas and endotoxin testing (Fig. 5B). These data allowed the process to be authorized by the French regulatory agency (EudraCT 2020-001287-28) for use in a first clinical trial assessing UC-MSCs in patients with coronavirus type 2-associated severe ARDS [13].

## Discussion

During the last years, MSCs emerge as a perspective for ATMPs’ development for various diseases [19]. Several tissue sources have been evaluated, but the BM is still the most commonly used. The aim of our project was to develop and qualify a manufacturing process of an ATMP composed of MSCs derived from UC that presents several advantages compared to other tissues (i.e., BM or AT). The UC represents an unlimited available tissue source, with few ethical and safety concerns [20]. Thus, it offers the possibility to produce an off-the-shelf medicinal product, ready to use for several patients, allowing to reduce delays compared to autologous products. Indeed, the manufacturing of MSCs therapeutic doses requires several weeks, which may be incompatible with the clinical status of the patient. In addition, UC-MSCs feature immunomodulatory functions purportedly stronger than those of MSCs derived from other sources [21], which make them particularly attractive for treating immune and/or inflammatory diseases.

During this project, we developed a process based on the isolation of MSCs from human UCs using the explant method, followed by their expansion in vitro for several passages. Among fifteen UCs, twelve were processed successfully allowing a high success rate. However, one of the complexities was the management of the variability between donors, making difficult to standardize the process. Indeed, the minimal quantity of isolated cells at P0 was 0.2 × 10^5^ cells and the maximum 20.3 × 10^5^ showing a variability of up to 2 log_10_ between donors. Likewise, the DT and PD varied by a factor of two between donors, ranging from 14.3 h to 24.4 h and from 5.9 to 10.5, respectively, between P0 and P1. The assessment of the long-term proliferation of UC-MSCs showed an important increase of DT and decrease of PD associated with a senescent morphology for later passages, thereby demonstrating the absence of MSCs stemness as previously described [22]. However, during early passages UC-MSCs displayed higher proliferation capacities than adult tissue-derived MSCs [23] due to their primitive state. We validated the P3 as the later passage for the clinical use of UC-MSCs since, at this stage, cells showed high proliferative capacities without chromosomal abnormalities.

To further characterize UC-MSCs, we assessed their biological properties and immunomodulatory activities on three batches derived from a single donor. An extended immunophenotype with the assessment of mesenchymal, immunogenic and co-stimulatory markers was performed. CD90, CD105, CD73, CD44, CD29 and CD166 were homogenously positive, whereas the hematopoietic and endothelial markers CD14, CD45 and CD31 were negative. Similarly to BM-MSCs and AD-MSCs, the class I HLA-ABC was positively expressed by UC-MSCs, while no expression of class II HLA-DR or co-stimulatory molecules CD40, CD80 and CD86 was observed [24]. Furthermore, in contrast to what has been described for BM-MSCs, HLA-DR expression was not induced in UC-MSCs after IFNγ treatment, suggesting a lower immunogenic profile of UC-MSCs for allogenic use in inflammatory diseases. However, the determination of the donors and recipients HLA typing still appears necessary since studies assessing immune responses after MSCs injection have shown that HLA-mismatched MSCs are not immunoprivileged [25]. In vitro, immunological assays can allow to evaluate MSCs immunogenicity, but they are still poorly predictive of in vivo alloreactivity, requiring a confirmation by in vivo assays [25]. Thus, the development of more robust and relevant in vitro assays is necessary to provide quality controls for assessing the potential immunogenicity of the cell product.

Interestingly, UC-MSCs’ morphology, proliferative capacities and phenotype were not modified after priming, suggesting that UC-MSCs are able to preserve their basal properties in a pro-inflammatory environment. The pericyte marker CD146 was moderately expressed and heterogeneous within cell populations. This variability may be explained by the UC-MSCs’ subsets localization in the UC, close to the blood vessels or not. Bowles et al. showed that CD146^+^ BM-MSCs display higher secretory profile and immunomodulatory properties compared to CD146^−^ subsets [26]. Pro-inflammatory priming enhanced CD146^+^ expression, which was correlated with the increase of BM-MSCs immunomodulatory properties. Our results showed that the CD146 expression by UC-MSCs reached its peak value at their basal state, thus not requiring further inflammatory stimulation. These results are concordant with the higher immunomodulatory potential of UC-MSCs compared to the gold-standard BM-MSCs [3]. It will be interesting to evaluate the CD146 expression by UC-MSCs depending on their anatomic localization and to compare the immunomodulatory properties of spatially distinct subsets.

To further characterize UC-MSCs' immunomodulatory properties, we performed potency assays to evaluate cell functionality at basal state and in pro-inflammatory conditions. UC-MSCs were treated with IFNγ, TNFα and the combination of IFNγ + TNFα according to the ISCT® recommendations [15]. In addition, we evaluated the impact of IL1β and IL6 largely described as mediators of inflammation and autoimmunity [9, 11], and GM-CSF, involved in chronic inflammation [12]. IDO has been demonstrated as particularly involved in the immunomodulatory functions of human MSCs [18]. Our results show that IDO was not expressed by UC-MSCs at basal state but was significantly enhanced after priming by IFNγ. However, treatment with other inflammatory cytokines did not induce IDO expression, which supports the key role of IFNγ for activation of the UC-MSCs’ immunomodulatory functions. Previous studies have identified several molecules as markers of MSCs activation related to their immunomodulatory capacities [27, 28]. In contrast to BM-MSCs [28], our results show that UC-MSCs expressed positively ICAM-1/CD54 at basal state. Moreover, under inflammatory conditions, ICAM-1/CD54 expression was increased significantly. In addition, treatment with INFγ + TNFα enhanced the expression of VCAM-1/CD106. ICAM-1 and VCAM-1 are involved in the recruitment of T-cells to inflammatory sites and the negative regulation of their proliferation through the cell-contact mechanism [27, 28]. Our results also show that UC-MSCs expressed positively the INFγ-Receptor/CD119 but not the TNFα-Receptor/CD120b even after priming, which may explain the absence of UC-MSCs immune activation with TNFα treatment.

It is important to notice that these results may vary depending on several factors including but not limited to culture conditions, priming conditions and analytical methods, tissue or species origin [15, 29]. Thus, to confirm UC-MSCs immunomodulatory properties, we performed a functional assay using a standardized and validated MLR-assay according to the ICH Q2(R1) [16]. The ability of UC-MSCs to inhibit T-Ly proliferation was observed without any pro-inflammatory treatment, even at low doses (ratio 1:100). These results confirm the high UC-MSCs immunomodulatory properties at basal state, in contrast to BM-MSCs that need inflammatory conditions [24]. Indeed, the comparison of UC-MSCs to BM-MSCs previously validated and prepared for another clinical trial (NCT02213705) showed that UC-MSCs inhibitory effect on T-Ly proliferation was significantly higher compared to BM-MSCs (AUC = 0,150 ± 0,006 *versus* 0,098 ± 0,007; *p* < 0.001, Welch’s test), supporting the higher immunomodulatory properties of UC-MSCs in vitro at basal state (Additional file 6). However, these preliminary results need to be confirmed in a greater cohort and under standardized experimental conditions. UC-MSCs pro-inflammatory treatment with IFNγ or IFNγ + TNFα enhanced significantly their inhibitory effect on T-Ly proliferation. Thus, the increase of marker expression associated with the decrease of T-Ly proliferation confirms the key role of IFNγ to potentiate UC-MSCs biological activity in vitro.

After the validation of UC-MSCs’ biological properties and activity, we transferred the technology and scale-up of the manufacturing process to a GMP facility in order to produce an UC-MSC-based ATMP for clinical use in immune and/or inflammatory diseases. Given the important heterogeneity between donors, we decided to develop a manufacturing process of an UC-MSC-based ATMP derived from a single donor, with a system of master and work cell stocks. Qualifications performed in process on the MCS and WCS allowed the approval of our process by the French regulatory agency for manufacturing an investigational UC-MSC-based cell therapy in the treatment of SARS-Cov-2-induced ARDS [13]. Because a key pathophysiological feature of this disease is an acute pulmonary inflammation, the aim was to reduce the inflammatory storm thanks to the UC-MSCs’ immunomodulatory properties. Our perspective is to enlarge this process to other immune and/or inflammatory diseases including but not limited to the GvHD or inflammation subsequent to traumatic injury.

It is important to highlight that our process was based on the use of an UC collected from a single donor. However, inter-donor heterogeneity has already been reported and may influence the therapeutic effect of MSC-based therapies leading to treatment failure [30]. In order to address this issue, our perspective is to first assess and compare UC-MSC properties and biological activities between several donors and to define specifications allowing to identify the best donors. Then, we will compare these properties with a pool of several donors, to assess biological properties and functions of pooled UC-MSCs.

## Conclusion

UC-MSCs are particularly attractive as they offer the possibility to generate cell banks and to produce off-the-shelf medicinal products, thereby allowing to reduce delays compared to autologous products and treat large patient cohorts. However, the heterogeneity between donors remains a challenge for manufacturing standardization and clinical efficacy anticipation. A stringent selection of « ideal» donors as well as a better knowledge of UC-MSCs mechanism of action should allow improving the development of UC-MSC-based ATMPs.

## Supplementary Information

**Additional file 1. **Serological testing according to the European directive on tissue procurement. * UC collected during the COVID-19 pandemic.

**Additional file 2. **Cytokines used to mimic the pro-inflammatory environment in vitro.

**Additional file 3. **Antibodies used for UC-MSCs staining at basal state and after pro-inflammatory priming.

**Additional file 4. **Summary of the manufacturing process steps: UC procurement, UC processing, UC removal, MSCs expansion until passage 2 (P2), MCS production, WCS manufacturing, ATMP manufacturing. P: passage, d: day.

**Additional file 5. **Negative controls of CD90, CD105, CD73, CD44, CD29, CD166, CD146, HLA-DR, HLA-ABC, CD40, CD80, CD86 markers expression. Fluorescence Minus One (FMO) expression of CD14, CD45, CD31 markers.

**Additional file 6. **Area under the curve (AUC) representing the UC-MSCs and BM-MSCs’ abilities to inhibit T-Lymphocyte proliferation, which allow to assess their immunomodulatory activity. P < 0.001, Welch’s test.

## Data Availability

All data generated or analyzed during this study are included in this published article [and its supplementary information files].

## Abbreviations

ATMP: Advanced therapy medicinal product
AT-MSCs: Adipose tissue-derived MSCs
BM: Bone marrow
BM-MSCs: Bone marrow-derived MSCs
CFU-F: Colony-forming unit-fibroblastic
CTV: CellTrace™ violet
DMSO: Dimethylsulfoxide
DPBS: Dulbecco’s phosphate-buffered saline
DT: Doubling time
EMA: European Medicines Agency
GM-CSF: Granulocyte macrophage colony-stimulating factor
GMP: Good manufacturing practices
GMP-ATMP: Good manufacturing practices specific to advanced therapy medicinal products
GvHD: Graft versus host disease
IDO: Indoleamine 2,3-dioxygenase
IFNγ: Interferon γ
IL: Interleukin
ISCT: International Society for Cell and Gene Therapy
MA: Marketing authorization
MCS: Master cell stock
MLR: Mixed lymphocyte reaction
MSCs: Mesenchymal stromal cells
MEM-α: Minimum essential medium α
PL: Platelet lysate
NT: Non-treated
P: Passage
PBMC: Peripheral blood mononuclear cells
PD: Population doubling
RPMI: Roswell Park Memorial Institute
T-Ly: T lymphocyte
TNFα: Tumor necrosis factor α
UC: Umbilical cord
UC-MSCs: Umbilical cord-derived mesenchymal stromal cells
WCS: Work cell stock
