# Supplementary material for: Development of a human umbilical cord-derived mesenchymal stromal cell-based advanced therapy medicinal product to treat immune and/or inflammatory diseases
Source: Stem Cell Res Ther. 2021 Nov 13;12:571. doi: 10.1186/s13287-021-02637-7 (PMC8590372; doi:10.1186/s13287-021-02637-7)
Supplement: Supplementary file 1 — Additional file 1. Serological testing according to the European directive on tissue procurement. * UC collected during the COVID-19 pandemic. [file 13287_2021_2637_MOESM1_ESM.pptx]

## Slide 1
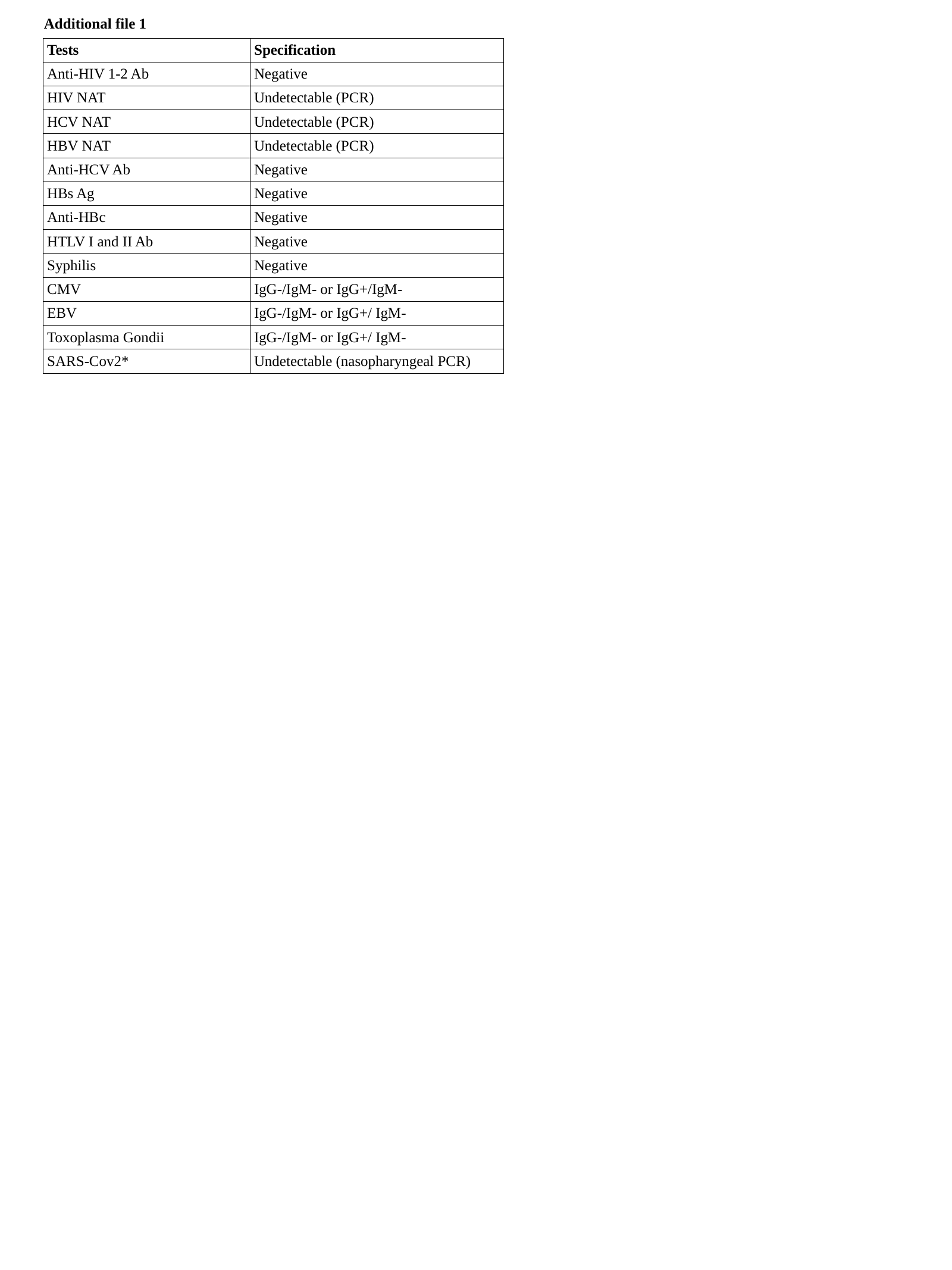

Additional file 1
| Tests | Specification |
| --- | --- |
| Anti-HIV 1-2 Ab | Negative |
| HIV NAT | Undetectable (PCR) |
| HCV NAT | Undetectable (PCR) |
| HBV NAT | Undetectable (PCR) |
| Anti-HCV Ab | Negative |
| HBs Ag | Negative |
| Anti-HBc | Negative |
| HTLV I and II Ab | Negative |
| Syphilis | Negative |
| CMV | IgG-/IgM- or IgG+/IgM- |
| EBV | IgG-/IgM- or IgG+/ IgM- |
| Toxoplasma Gondii | IgG-/IgM- or IgG+/ IgM- |
| SARS-Cov2\* | Undetectable (nasopharyngeal PCR) |
