# Supplementary material for: Development of a human umbilical cord-derived mesenchymal stromal cell-based advanced therapy medicinal product to treat immune and/or inflammatory diseases
Source: Stem Cell Res Ther. 2021 Nov 13;12:571. doi: 10.1186/s13287-021-02637-7 (PMC8590372; doi:10.1186/s13287-021-02637-7)
Supplement: Supplementary file 2 — Additional file 2. Cytokines used to mimic the pro-inflammatory environment in vitro. [file 13287_2021_2637_MOESM2_ESM.pptx]

## Slide 1
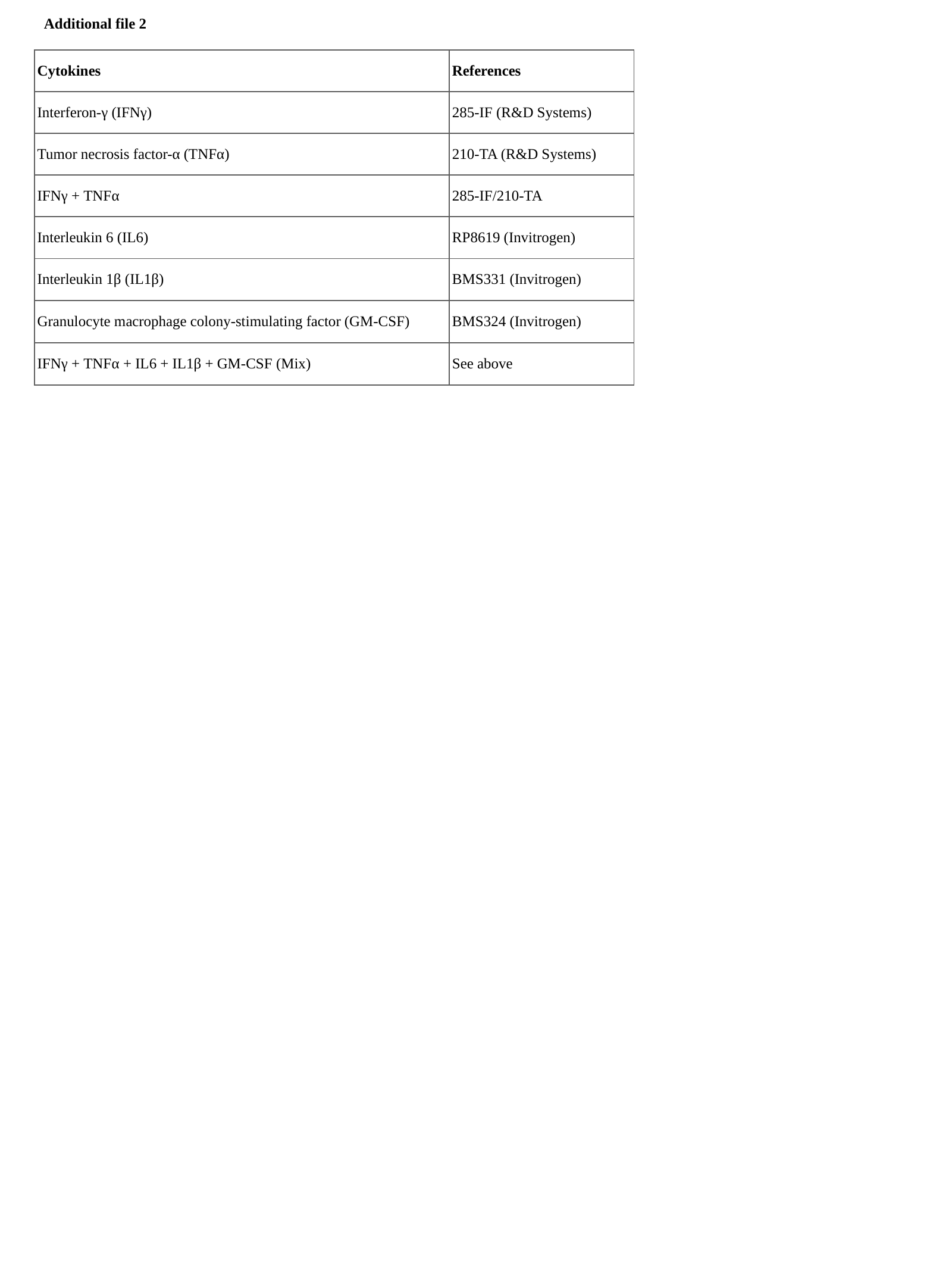

Additional file 2
| Cytokines | References |
| --- | --- |
| Interferon-γ (IFNγ) | 285-IF (R&D Systems) |
| Tumor necrosis factor-α (TNFα) | 210-TA (R&D Systems) |
| IFNγ + TNFα | 285-IF/210-TA |
| Interleukin 6 (IL6) | RP8619 (Invitrogen) |
| Interleukin 1β (IL1β) | BMS331 (Invitrogen) |
| Granulocyte macrophage colony-stimulating factor (GM-CSF) | BMS324 (Invitrogen) |
| IFNγ + TNFα + IL6 + IL1β + GM-CSF (Mix) | See above |
