# Supplementary material for: Development of a human umbilical cord-derived mesenchymal stromal cell-based advanced therapy medicinal product to treat immune and/or inflammatory diseases
Source: Stem Cell Res Ther. 2021 Nov 13;12:571. doi: 10.1186/s13287-021-02637-7 (PMC8590372; doi:10.1186/s13287-021-02637-7)
Supplement: Supplementary file 3 — Additional file 3. Antibodies used for UC-MSCs staining at basal state and after pro-inflammatory priming. [file 13287_2021_2637_MOESM3_ESM.pptx]

## Slide 1
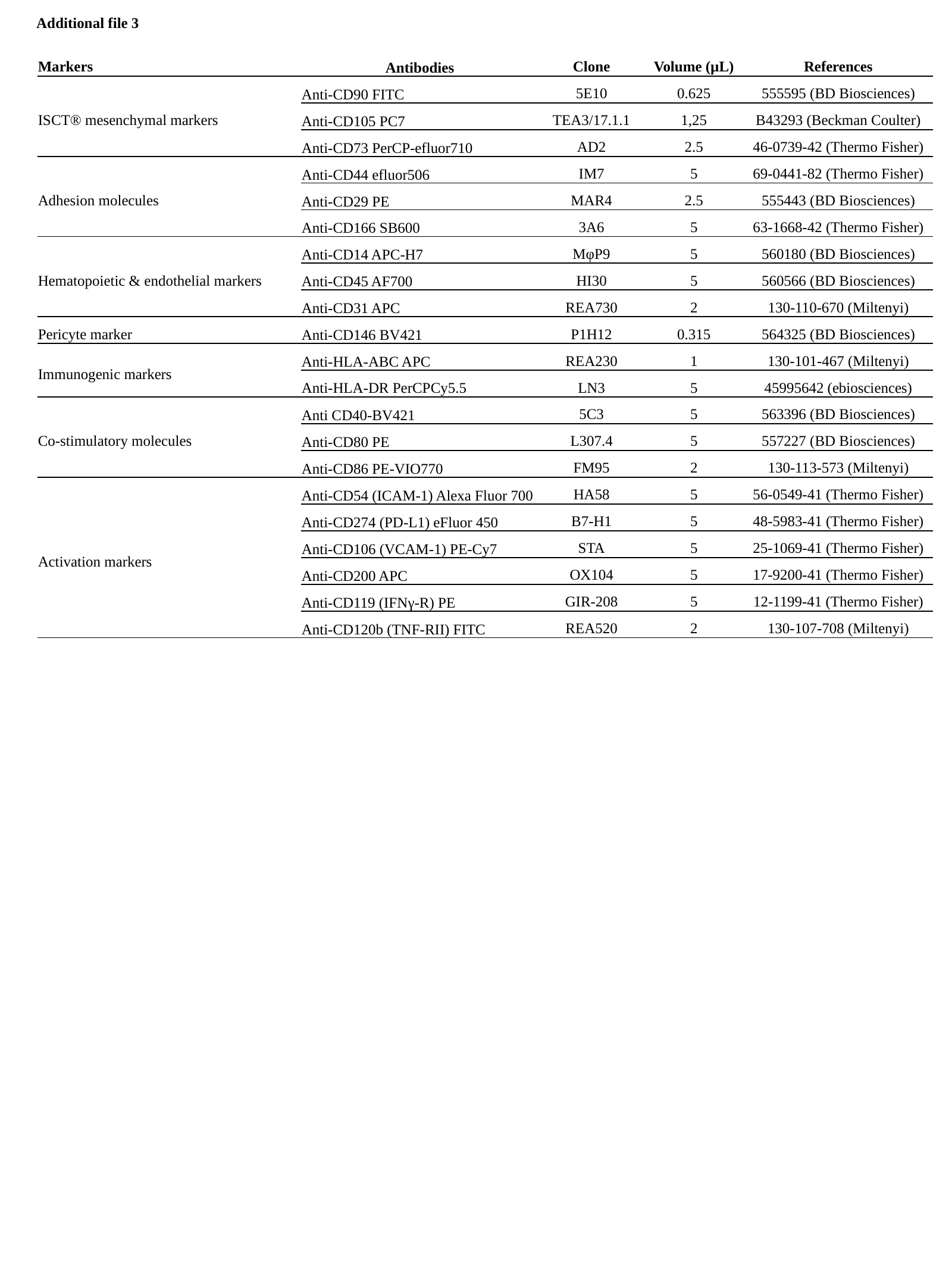

Additional file 3
| Markers | Antibodies | Clone | Volume (µL) | References |
| --- | --- | --- | --- | --- |
| ISCT® mesenchymal markers | Anti-CD90 FITC | 5E10 | 0.625 | 555595 (BD Biosciences) |
| | Anti-CD105 PC7 | TEA3/17.1.1 | 1,25 | B43293 (Beckman Coulter) |
| | Anti-CD73 PerCP-efluor710 | AD2 | 2.5 | 46-0739-42 (Thermo Fisher) |
| Adhesion molecules | Anti-CD44 efluor506 | IM7 | 5 | 69-0441-82 (Thermo Fisher) |
| | Anti-CD29 PE | MAR4 | 2.5 | 555443 (BD Biosciences) |
| | Anti-CD166 SB600 | 3A6 | 5 | 63-1668-42 (Thermo Fisher) |
| Hematopoietic & endothelial markers | Anti-CD14 APC-H7 | MφP9 | 5 | 560180 (BD Biosciences) |
| | Anti-CD45 AF700 | HI30 | 5 | 560566 (BD Biosciences) |
| | Anti-CD31 APC | REA730 | 2 | 130-110-670 (Miltenyi) |
| Pericyte marker | Anti-CD146 BV421 | P1H12 | 0.315 | 564325 (BD Biosciences) |
| Immunogenic markers | Anti-HLA-ABC APC | REA230 | 1 | 130-101-467 (Miltenyi) |
| | Anti-HLA-DR PerCPCy5.5 | LN3 | 5 | 45995642 (ebiosciences) |
| Co-stimulatory molecules | Anti CD40-BV421 | 5C3 | 5 | 563396 (BD Biosciences) |
| | Anti-CD80 PE | L307.4 | 5 | 557227 (BD Biosciences) |
| | Anti-CD86 PE-VIO770 | FM95 | 2 | 130-113-573 (Miltenyi) |
| Activation markers | Anti-CD54 (ICAM-1) Alexa Fluor 700 | HA58 | 5 | 56-0549-41 (Thermo Fisher) |
| | Anti-CD274 (PD-L1) eFluor 450 | B7-H1 | 5 | 48-5983-41 (Thermo Fisher) |
| | Anti-CD106 (VCAM-1) PE-Cy7 | STA | 5 | 25-1069-41 (Thermo Fisher) |
| | Anti-CD200 APC | OX104 | 5 | 17-9200-41 (Thermo Fisher) |
| | Anti-CD119 (IFNγ-R) PE | GIR-208 | 5 | 12-1199-41 (Thermo Fisher) |
| | Anti-CD120b (TNF-RII) FITC | REA520 | 2 | 130-107-708 (Miltenyi) |
