# Supplementary figures and images for: Development of a human umbilical cord-derived mesenchymal stromal cell-based advanced therapy medicinal product to treat immune and/or inflammatory diseases
Source: Stem Cell Res Ther. 2021 Nov 13;12:571. doi: 10.1186/s13287-021-02637-7 (PMC8590372; doi:10.1186/s13287-021-02637-7)

## Slide 1
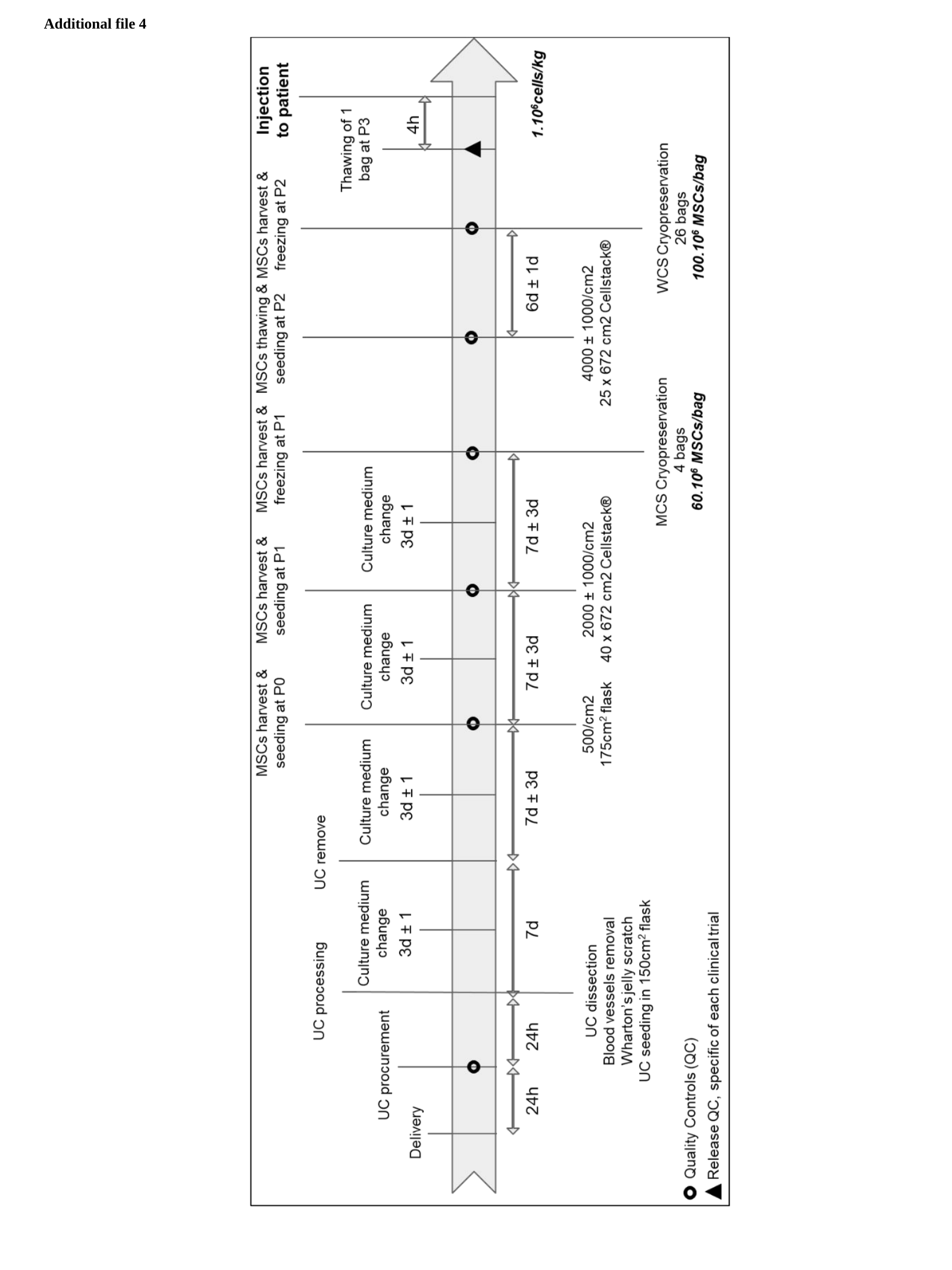

Additional file 4

Supplement: Supplementary file 4 — Additional file 4. Summary of the manufacturing process steps: UC procurement, UC processing, UC removal, MSCs expansion until passage 2 (P2), MCS production, WCS manufacturing, ATMP manufacturing. P: passage, d: day. [file 13287_2021_2637_MOESM4_ESM.pptx]

## Slide 1
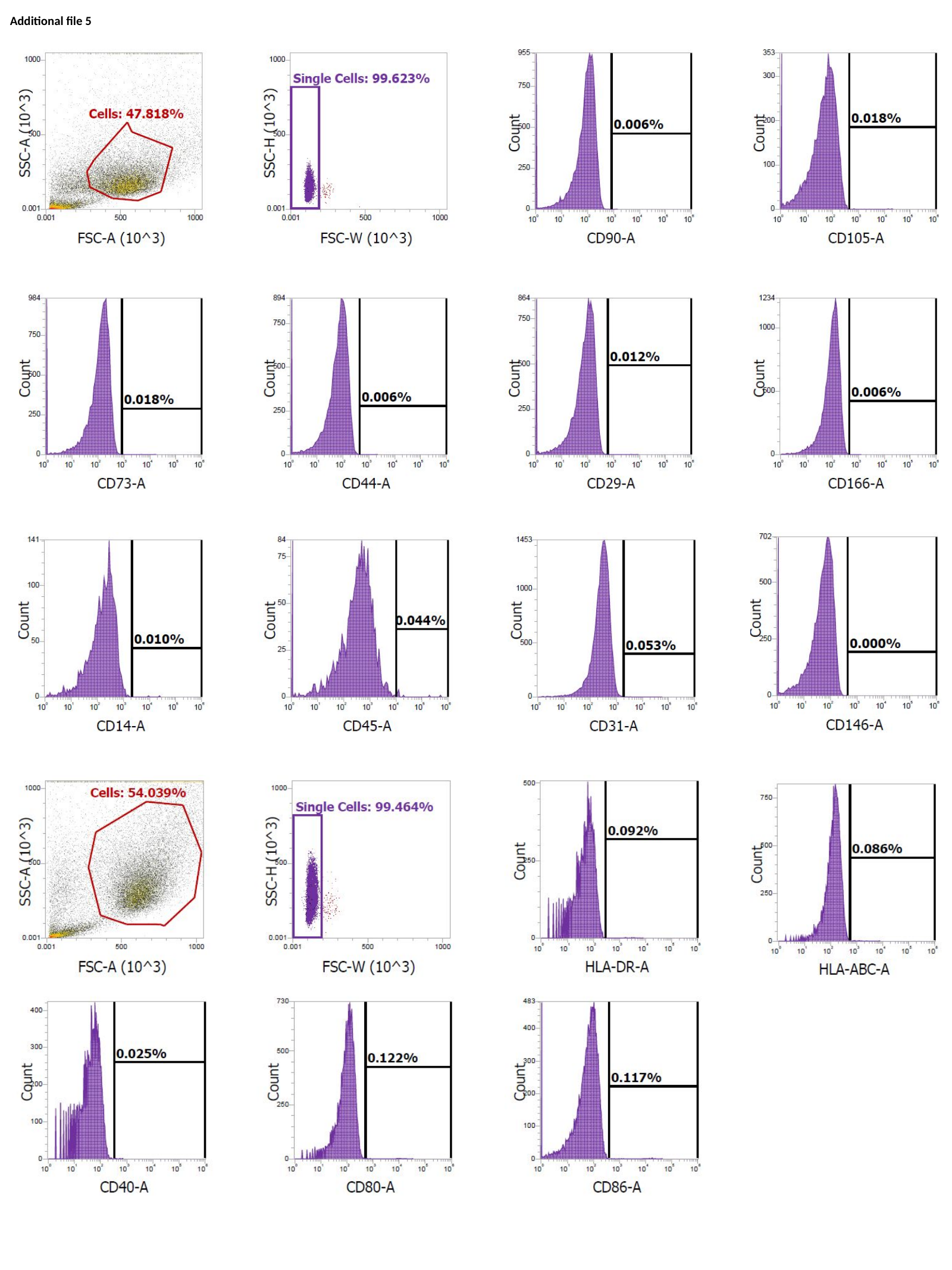

Additional file 5

Supplement: Supplementary file 5 — Additional file 5. Negative controls of CD90, CD105, CD73, CD44, CD29, CD166, CD146, HLA-DR, HLA-ABC, CD40, CD80, CD86 markers expression. Fluorescence Minus One (FMO) expression of CD14, CD45, CD31 markers. [file 13287_2021_2637_MOESM5_ESM.pptx]

## Slide 1
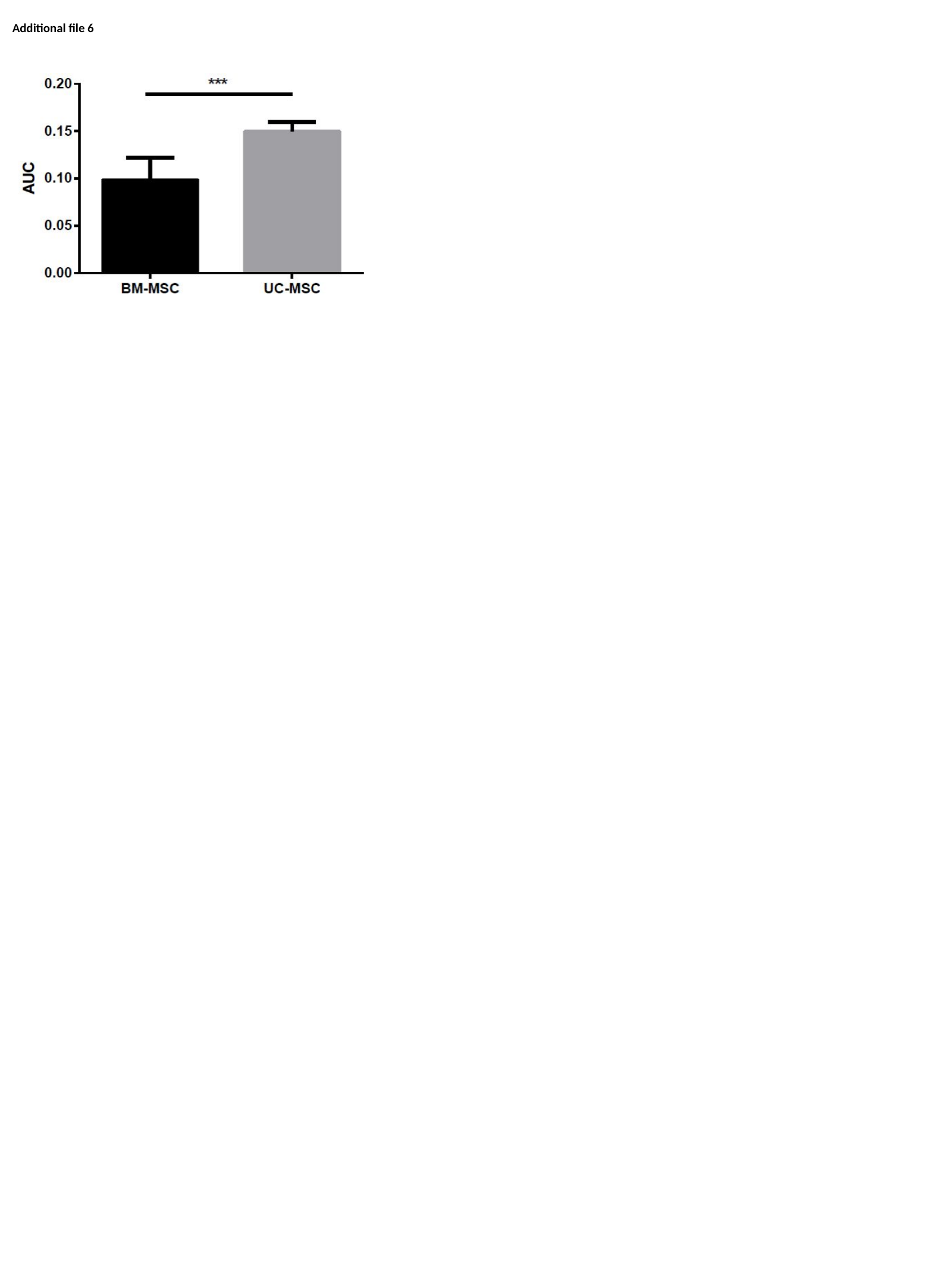

Additional file 6

Supplement: Supplementary file 6 — Additional file 6. Area under the curve (AUC) representing the UC-MSCs and BM-MSCs’ abilities to inhibit T-Lymphocyte proliferation, which allow to assess their immunomodulatory activity. P < 0.001, Welch’s test. [file 13287_2021_2637_MOESM6_ESM.pptx]
